# An epigenetic mechanism for differential maturation of amygdala–prefrontal connectivity in childhood socio-emotional development

**DOI:** 10.1038/s41398-023-02380-y

**Published:** 2023-03-13

**Authors:** Amalia M. Skyberg, Benjamin T. Newman, Andrew J. Graves, Alison M. Goldstein, Samantha R. Brindley, Minah Kim, T. Jason Druzgal, Jessica J. Connelly, James P. Morris

**Affiliations:** 1 University of Virginia, Department of Psychology, 102 Gilmer Hall, P.O. Box 400400, Charlottesville, VA 22904 USA; 2 University of Virginia, School of Medicine, Department of Radiology and Medical Imaging, 1215 Lee St, Charlottesville, VA 22908 USA

**Keywords:** Epigenetics and behaviour, Human behaviour

## Abstract

Functional connectivity between the amygdala and the medial prefrontal cortex (mPFC) has been identified as a neural substrate of emotion regulation that undergoes changes throughout development, with a mature profile typically emerging at 10 years of age. Maternal bonding in childhood has been shown to buffer amygdala reactivity and to influence the trajectory of amygdala–mPFC coupling. The oxytocinergic system is critical in the development of social behavior and maternal bonding. Early-life parental care influences the methylation status of the oxytocin receptor (*OXTR*m) in animal models and humans, and higher *OXTR*m is associated with lower amygdala–PFC functional connectivity in adults. Using a neuroimaging-epigenetic approach, we investigated saliva-derived *OXTR*m as a biological marker of structural and functional connectivity maturation in 57 typically developing children (*P* < 0.05). We utilized seed-based connectivity analysis during a novel abstract movie paradigm and find that higher levels of *OXTR*m are associated with a more adult-like functional connectivity profile. Concurrently, more adult-like functional connectivity was associated with higher reported self-control and more diffusion streamlines between the amygdala and mPFC. *OXTR*m mediates the association between structural and functional connectivity with higher levels of *OXTR*m being associated with more streamlines. Lastly, we also find that lower *OXTR*m blunts the association between amygdala–mPFC connectivity and future internalizing behaviors in early adolescence. These findings implicate *OXTR*m as a biological marker at the interface of the social environment and amygdala–mPFC connectivity in emotional and behavioral regulation. Ultimately, identification of neurobiological markers may lead to earlier detection of children at risk for socio-emotional dysfunction.

## Introduction

Emotion regulation is an essential component of social development that allows individuals to successfully form relationships and find success at school, work, and home. Deficits in emotion regulation have been associated with several internalizing psychopathologies, including depression [1] and anxiety disorders [2]. A well-characterized neural substrate of emotion regulation is functional connectivity between amygdala and frontal cortical regions, specifically medial prefrontal cortex (mPFC) [3, 4]. Accumulating evidence suggests that the prefrontal cortex may regulate the amygdala and enable successful regulation of emotion and behavior through a top-down mechanism [5]. Importantly, both human and animal studies suggest that the strength and direction of amygdala–mPFC functional connectivity change throughout development [6–8] and these changes can be altered by early-life experience [9–11].

Age-related changes associated with functional and structural connectivity between amygdala and mPFC are characterized by a pronounced shift during early adolescence, or around age 10–11 years old [6, 7, 12]. Functional neuroimaging studies have demonstrated a decline in amygdala reactivity and an increase in prefrontal activation with age as children transition to adolescence [7, 8, 13]. Similarly, functional connectivity between the amygdala and mPFC during emotional tasks shifts from positive functional connectivity in childhood to negative functional connectivity in adolescence, which more closely fits the mature top-down regulatory profile proposed for the circuit in adults with negative functional connectivity [7]. However, the strength and valence of amygdala–prefrontal connectivity during resting-state paradigms is less consistent, with some studies reporting an increase in amygdala–mPFC connectivity with age [6, 14] and others reporting a decrease in amygdala–mPFC connectivity with age [15, 16]. Discrepancies in these findings may be due to localization specificity of mPFC regions, but nonetheless, prior research indicates an acute shift in amygdala–mPFC connectivity occurs between childhood, adolescence, and young adulthood.

A prevailing hypothesis suggests that there is a sensitive period during development in which early-life experience may alter the normative protracted development of this neural circuit. Young children raised with consistent access to a caregiver may utilize the caregiver to buffer amygdala reactivity while amygdala–PFC functional connectivity is still immature [17]. On the contrary, early-life stress or the absence of reliable caregiver presence may accelerate the trajectory of amygdala–prefrontal functional connectivity [18–20]. In studies comparing previously institutionalized youth to age-matched typically reared peers, previously institutionalized children exhibit a more negative (or “adult-like”) profile of connectivity at an earlier age [18]. This mature profile is associated with more successful emotion regulation during childhood, perhaps acting as an adaptive, compensatory mechanism for early-life stress in the absence of a caregiver who would normally attenuate amygdala response. Adolescents with other forms of early-life stress, such as a history of childhood trauma, also show weaker resting-state amygdala–prefrontal functional connectivity [21, 22] and are at an increased risk for internalizing psychopathology later in life [21].

Early-life experience can alter the trajectory of amygdala–mPFC functional connectivity, but the specific biological mechanisms that contribute to the establishment, fine-tuning, and maintenance of this system remain unknown. The oxytocinergic system has been implicated as a key modulator of emotion regulation in both animals [23] and humans [24]. Manipulation of oxytocin levels both endogenously and exogenously indicates that this system may help this circuit respond and adapt to arousal. The precise action of oxytocin is dependent upon the expression of its receptor, which is encoded by the oxytocin receptor gene (*OXTR*). Expression of *OXTR* is epigenetically regulated such that DNA methylation of *OXTR* (*OXTR*m) is associated with reduced expression of *OXTR* in the human cortex [25]. There is convincing evidence that peripheral measurements of *OXTR*m may be used as a marker of the activity and DNA methylation state of this gene in the human brain [25] and in animal model systems [26]. In addition, recent work has demonstrated that blood and saliva-derived methylation levels are highly correlated in humans, allowing non-invasive collection in developmental populations where an intravenous blood draw is less feasible [27].

Evidence from animals and humans suggests that natural variation in parental care without extreme circumstances may also have lasting impacts on neural and behavioral differences across the lifespan [28, 29]. Research in the prairie vole animal model has revealed that levels of *OXTRm* are sensitive to variability in social environment and quality of parental care [26]. High-care parenting in the first week of life prevents de novo DNA methylation of *OXTR* in juveniles and leads to increases in species typical social behavior. Additionally, offspring of low-care parents display faster rates of development on several physical markers, such as eye-opening, leaving the nest, and eating solid food, and these effects are likely mediated by *OXTR*m [29]. Recent work in human infants revealed a similar association between early-life maternal care and change in the methylation status of *OXTR*. Specifically, saliva-derived *OXTR*m is dynamic in infancy between 5 and 18 months of age [30] and changes in DNA methylation levels were associated with maternal engagement, where higher maternal engagement predicted a reduction in infant *OXTR*m from 5 to 18 months [30]. These data in both animals and humans suggest that parental care influences the DNA methylation status of *OXTR*, at least in early life, which may have implications on the trajectory of social development. In addition to its associations with variable social environments, human neuroimaging studies have identified *OXTR*m as a molecular correlate of neural emotional processing. In adults, higher levels of *OXTR*m are associated with increased amygdala reactivity to fearful and angry faces and weaker amygdala–frontal cortical coupling during an emotional face task [31].

Taken together, *OXTR*m is a particularly well-suited biological marker to investigate differential maturation of amygdala–mPFC functional connectivity given that *OXTR*m is associated with amygdala–mPFC connectivity in adults and affected by the social environment. We investigated *OXTR*m as a biological marker for neural and behavioral trajectories of social maturation in children 5–11 years old in a prospective behavioral longitudinal study. Specifically, we hypothesized that irrespective of chronological age, higher levels of *OXTR*m would be associated with a more adult-like and mature amygdala–frontal cortical functional connectivity profile during a naturalistic viewing resting-state paradigm. We further hypothesize that *OXTR*m would be associated with structural connectivity between the amygdala and mPFC. Lastly, we hypothesized that amygdala–mPFC functional connectivity and *OXTR*m would be associated with concurrent emotion regulation abilities and predictive of internalizing symptomatology 2 years later.

## Materials and methods

### Study design

Seventy-six typically developing children between the ages of 5–11 years old were recruited from the local community for the current longitudinal study. Families were identified by responding to flyers or by having previously signed up to be contacted for developmental research studies. The funding award for this study with the UVA Brain Institute was for 60 children based on the power analysis conducted for the research proposal. An additional 31 adults (17 female, *M* = 28.02, SD = 1.65) aged 25–30 years old were enrolled in a separate neuroimaging study and completed the same fMRI *Inscapes* paradigm as the children and provided a saliva sample. Only self-reported Caucasians were recruited because population stratification can be a major issue for genetic association studies where systematic differences in allele frequencies across populations can lead to spurious associations[32]. Additional exclusion criteria for this study included the use of psychotropic medications and MRI contraindications. Informed written consent was obtained from the adult participant or legal guardian of each child, and participants independently assented to study participation for protocols approved by the University of Virginia Institutional Review Board.

Participation for the children consisted of up to four study visits over 2 years. The first timepoint was broken up into two study visits spaced about three weeks apart (*M* = 21.76 days, SD = 12.69). At the first visit, children completed a 15-min mock scan to acclimate to the scanner environment and practice lying still. Participants who tolerated the mock scan were then scheduled for an MRI visit. Toleration of the scanning environment was determined by participants’ affect and comfort in the mock scan as well as their assent to participate in the real MRI scan. Participants who became distressed and did not tolerate the mock scan environment did not differ in sample characteristics from those who did in terms of age, sex, or parental education (all *P* > 0.05). Saliva was collected at the time of scanning at the second visit. Families were compensated $50 at each visit and the child received a toy at each visit. Adults were compensated $50 for their participation in the neuroimaging study. A total of 16 child participants and one adult participant were excluded because of excessive head motion, and one adult reported falling asleep during the scan. Epigenotyping from three participants failed pyrosequencing, resulting in a final sample of 57 children (32 males, *M* = 8.15 y, SD = 1.57) with *OXTR*m and functional brain imaging data. Of these 57 participants, six did not complete the diffusion-weighted image sequence after the functional data was collected.

All children were contacted to participate in follow-up behavioral timepoints one and 2 years after completing the MRI. Children over the age of 8 years completed self-reported questionnaires. Due to the COVID-19 pandemic, some of these visits were conducted remotely when in person data collection was not permitted or preferred. We arranged to drop off study materials at the participants’ homes and study visits were conducted over Zoom (Zoom Video Communications Inc., 2016) to ensure consistency and data accuracy. Questionnaires were completed on an iPad Pro dropped off at the participants home and used Join.me (https://www.join.me/) to enable screen sharing so the rater could assist and monitor data collection.

### Parent-report demographic and behavioral data

Parent education was assessed via a brief demographic questionnaire to indicate the highest degree completed by the child’s caregiver(s) and for all participants degree completion of two caregivers were provided. For each participant, parent education was scored from 0 (8th grade) to 5 (PhD or other terminal degrees) and summed for both caregivers to generate a parent education value (*M* = 6.63, SD = 2.03, range = 2–10). Between the two visits at the first timepoint, caregivers were emailed a link to complete the Social Skills Improvement System rating scale (SSIS) [33] via Qualtrics [34]. The SSIS is a 79-item parent-report questionnaire designed to assess social skills and problem behaviors in children 3–18 years of age. Each item is rated on a 4-point Likert-type scale from 1 (never) to 4 (almost always). The self-control subscale was selected as the variable as interest as it most closely relates to emotion regulation abilities. Seven caregivers of participants included in the MRI analysis did not complete the questionnaires.

Two years later, children completed the student version of the SSIS. The student version is a 76-item self-report questionnaire designed to assess social skills and problem behaviors in children 8–18 years of age. Each domain is rated on a 4-point Likert-type scale from 1 (not very true) to 4 (very true). Of the 51 children that returned for the 2-year follow-up, six did not have neural or epigenetic data, lending a final sample size of 45 children.

### Saliva collection and epigenotyping procedures

Saliva was collected using CS-2 sponges in OG-250 collection kits from DNA Genotek (Ottawa, Canada) and stored at room temperature. Prior to DNA isolation, samples were incubated at 50 °C for 1 h and centrifuged at 1000 RPM for 10 min to recover all liquid from sponges. DNA was isolated following the manual purification protocol from DNA Genotek, resuspended in Hydration Solution (Qiagen, Hilden, Germany), and quantified using NanoDrop. Analysis of DNA methylation at CpG site −934 in *OXTR* was performed as previously reported in ref. [35] and are provided in the Supplementary Materials.

### Imaging procedures

Scanning was performed at the UVA Fontaine Research Park on a 3 T Siemens Prisma scanner with a 32-channel headcoil. Cushions were placed around the participants’ ears and forehead to minimize head movement during the scans. For the child participants, an experimenter stood by the participants’ feet to ensure that the participants remained awake and comfortable. If the experimenter noticed the participant move, they gently placed their hand on the participant’s leg to remind the participant to stay still.

Stimuli were presented with PsychoPy [36] using an LCD AVOTEC projector onto a screen located behind the subject’s head and viewed through an integrated headcoil mirror. In order to reduce the likelihood of head motion, participants viewed a 7-min movie paradigm, *Inscapes*, that features abstract shapes and music, but no narrative or social content. Audio was played at full volume inside the scanner room rather than through headphones to account for varying degrees of reported discomfort during piloting in the MRI-compatible headphone system. Research has shown that functional connectivity during *Inscapes* more closely resembles functional connectivity at rest than during a conventional movie clip, suggesting that it improves motion compliance while reducing cognitive load and therefore may be used as a proxy for the traditional resting-state paradigm [37].

### fMRI and diffusion analysis

Image acquisition and complete processing pipeline details for functional and diffusion scans are described in detail in the Supplementary Materials. For the *Inscapes* clip, seed-based correlation analysis was conducted using FSL’s general linear model (GLM) setup. Based on previous adult research with *OXTR*m as well as previously reported developmental finding, our primary hypotheses focus on the right amygdala as a seed region. We then conducted exploratory analyses using the left amygdala as a seed region. The left and right amygdala seed regions were selected from FSL’s Harvard–Oxford Subcortical atlas with a probabilistic threshold = 75%. Five mean-centered regressors were included in the model: a group mean regressor, *OXTR*m, age in months, sex, and parent education. We conducted an ROI analysis targeting the medial frontal cortex to identify regions in which amygdala–frontal cortical connectivity were positively or negatively significantly associated with *OXTR*m. Nonparametric statistics were utilized using FSL’s randomize and threshold-free cluster enhancement (TCFE [38]) to generate voxel-wise output from the general linear model within the non-thresholded mask of the Harvard–Oxford bilateral medial prefrontal cortex (5000 permutations, family-wise error *P* < 0.05).

For the diffusion scans, images were preprocessed using established methods to remove artifacts [39], the white matter fiber orientation distribution was resolved using single-shell constrained spherical deconvolution [40], and a whole-brain connectome was created using probabilistic tractography and the AAL atlas parcellation [41, 42]. Tracts from each participant’s connectome were used in the analysis if they terminated in the right or left amygdala and in the pooled ROIs that were selected if they overlapped the search region defined by the significant cluster in the fMRI analysis.

### Statistical modeling

Significant prefrontal clusters associated with the *OXTR*m regressor were registered to each participant’s native space, and average Z statistic values for each individual were extracted. We used the extracted Z statistic for right amygdala functional connectivity in the significant region from the *OXTR*m regressor for each individual to test how *OXTR*m and amygdala–mPFC connectivity might influence children’s behavior. All statistical analysis was conducted in R Studio [43], and code is available upon request. All models included age, sex, and parent education as covariates and models with structural streamline connectivity also included total brain volume as an additional covariate, as is standard in the field.

In order to model self-reported internalizing behaviors as a function of amygdala–mPFC functional connectivity, *OXTR*m, amygdala–mPFC streamlines, and demographics, we fit a Bayesian generalized linear model with a Skew-Normal likelihood. We used the Skew-Normal distribution as an alternative to the traditional Gaussian likelihood, because the response variable was slightly skewed to the lower portion of the scale (positive skew = 0.65, *P* = 0.029) [44]. Interpreting the coefficients of Skew-Normal regression is similar to traditional regression.

We used the *brms* software package in R [45] and the Hamiltonian Monte-Carlo No-U-Turn sampler (NUTS) for Bayesian computation and inference [46]. The primary motivation for taking a Bayesian approach here is the application of regularization priors to the regression coefficients as a way to employ shrinkage and mitigate potential for over-fitting generated by β ~*N* (*μ* = 0, σ = 10). Overall, this generally leads to more conservative and robust inference relative to the Frequentist approach [47]. This is particularly true when the sample size is relatively small. Default priors on the intercept were generated by Student *T* (*ν* = 3, *μ* = 6, σ = 4.4), the σ scale parameter generated by Student *T* (*ν* = 3, *μ* = 0, σ = 4.4), and the α shape parameter generated by *N* (*μ* = 0, σ = 4). We ran seven independent Markov chains each with 8000 total iterations, including 4000 warm-up iterations. We fixed the target average proposal acceptance probability to 99% to improve the quality of sampling and thus the resulting posterior distributions. Convergence of the posteriors were confirmed with all R̂ ≈ 1.0, which assesses agreement across the Markov chains.

## Results

A total of 57 typically developing self-identified Caucasian children aged 5–11 years old participated in this study. *OXTR*m was analyzed at CpG site -934, a CpG site within a region of the gene that has been shown to impact gene expression in a DNA methylation-dependent manner [48]. Saliva-derived DNA methylation at this site is highly correlated with blood-derived methylation [49] and has been associated with individual differences in amygdala response and functional connectivity to cortical opercular regions during an emotional face task in healthy adults [31]. At this site, *OXTR*m ranged from 36 to 54% methylated (*M* = 45.06, SD = 4.59). There were no significant associations of *OXTR*m with demographic control variables. See Supplementary Materials for additional descriptive statistics of variables.

### Cross-sectionally, amygdala–prefrontal connectivity is negatively associated with age

**Fig. 1:**
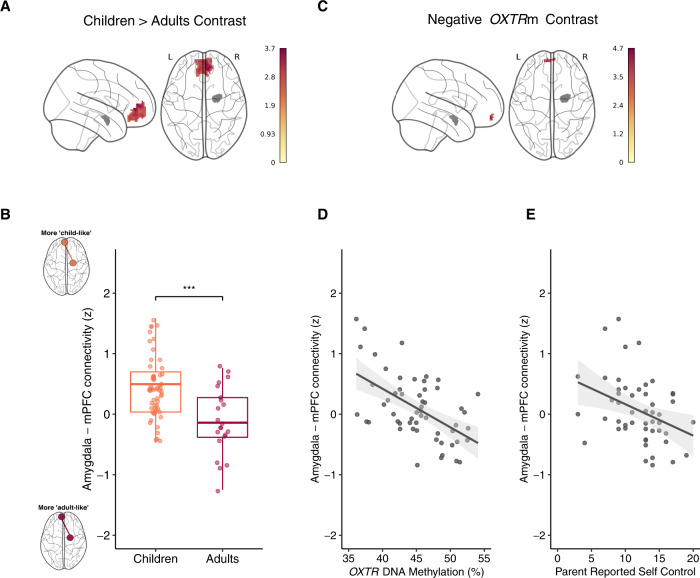
Adults show reduced right amygdala–mPFC functional connectivity compared to children. Children with increased methylation of OXTR display reduced right amygdala-mPFC functional connectivity. **A** Z statistic map of voxels shows the significant main effect of age group (Children > Adults) in MNI space. The right amygdala seed region is depicted in gray. **B** Mean *Z* statistic values are plotted for each individual in the child cohort (*n* = 57) and adult (*n* = 23) cohort. ***P < 0.001. **C** Z statistic map of voxels shows the significant negative main effect of *OXTR*m depicted in MNI space. The right amygdala seed region is depicted in gray. **D** Mean *Z* statistic values are plotted against percent *OXTR*m for each participant (*n* = 57). Gray shading indicates 95% confidence interval around the best-fit line. **E** Mean *Z* statistic values are plotted against parent-reported self-control scores for each participant (*n* = 50). Gray shading indicates 95% confidence interval around the best-fit line.

As previous studies examining the developmental shift in amygdala–prefrontal functional connectivity have been conducted using task-based fMRI or traditional rest paradigms, we first examined the developmental trajectory of this circuit between children and a small sample of adults. Using the right amygdala, we conducted a seed-based functional connectivity analysis to assess the effect of age on connectivity within the medial prefrontal cortex as defined by the Harvard–Oxford Atlas. This functional connectivity analysis revealed a significant main effect of age on the right (see Fig. 1A, B) and left (Supplementary Fig. S1) amygdala–prefrontal cortex connectivity such that adults exhibited less functional coupling during the passive viewing of *Inscapes*. Peak activity (*Z* = 3.73) for this cluster of voxels (*k* = 531) occurred at *x* = −8, *y* = −45, z = 1.

### Cross-sectionally, children have lower *OXTR* DNA methylation levels than young adults

We conducted an *t* test on all available samples to determine if adults and children differ in *OXTR*m levels. There was a significant effect of age cohort such that adults (*M* = 47.58, SD = 6.21) have a higher level of methylation at site –934 than children (*M* = 44.36, *SD* = 5.28), *t*(51) = −2.49, *P* = 0.016. See Supplementary Fig. S2 for visualization of mean methylation levels. However, within each cohort, there were no significant correlations with age (*P* > 0.05).

### *OXTR* DNA methylation is negatively associated with functional connectivity between the amygdala and mPFC

Using the right amygdala, we conducted a seed-based functional connectivity analysis to assess the effect of *OXTR*m on coupling within the medial prefrontal cortex as defined by the Harvard–Oxford Atlas while controlling for age, sex, and parent education. This analysis revealed a significant negative main effect of *OXTR*m on right amygdala functional connectivity with the left medial prefrontal cortex (see Fig. 1C, D). Peak activity (*Z* = 4.71) for this cluster of voxels (*k* = 36) occurred at *x* = −9, *y* = −57, *z* = −14. Exploratory analysis using the left amygdala seed region revealed no significant voxels.

### A more “adult-like” amygdala–mPFC functional connectivity profile is associated with more parent-reported self-control in social situations

Next, we tested the hypothesis that a more adult-like amygdala–mPFC functional connectivity profile would account for individual differences in concurrent emotion regulation and self-control behaviors. The *OXTR*m-defined mPFC cluster was registered to subject space, and the mean *Z* statistic value was extracted for each participant and correlated with scores from the SSIS self-control scale. We used linear models to examine the association between self-control behaviors and amygdala–mPFC connectivity with participant sex, age, and parent education included as covariates. Amygdala–mPFC functional connectivity had a significantly negative relationship with the self-control subscale (*B* = −2.73, *P* < 0.01, *η*_*p*_^*2*^ = 0.14) (Fig. 1E). None of the other covariates were significant.

### *OXTR* DNA methylation is associated with increased diffusion tractography streamlines between the amygdala and mPFC

**Fig. 2:**
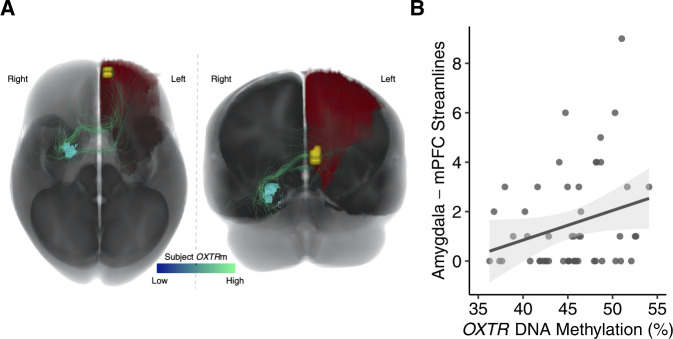
Individuals with increased methylation of *OXTR* show greater right amygdala–mPFC structural connectivity. **A** Visualization of total child cohort axonal connections between right amygdala (light blue) and the left frontal cortex ROI (red) while also displaying the location of the significant cluster determined from the fMRI analysis seeded in the right amygdala (yellow). Connections were visualized from the whole-brain tractograms of all subjects in the study and streamlines were colored based on the *OXTR*m of the associated subject, with darker blue signifying lower *OXTR*m and lighter green signifying higher *OXTR*m. **B** Number of streamlines are plotted against the percent *OXTR*m for each participant (*n* = 51). Gray shading indicates 95% confidence interval around the best-fit line.

A linear model was constructed to examine the association between *OXTR*m and the number of diffusion tractography streamlines in each subject connecting the right amygdala to the frontal cortex ROI defined by the fMRI search area. Participant sex, age, and brain volume were included as covariates. The number of streamlines connecting the right amygdala to the left frontal cortex ROI had a significantly positive relationship with *OXTR*m (*B* = 0.134, *P* < 0.05, *η*_*p*_^*2*^ = 0.09). See Fig. 2 for visualization of the number of streamlines associated with *OXTR*m levels. None of the other covariates had a significant relationship with the number of streamlines connecting the right amygdala to the left frontal cortex ROI. The connections between the left amygdala and the left frontal cortex ROI did not display a significant relationship between the number of streamlines and *OXTR*m.

### *OXTR* DNA methylation mediates the association between structural and functional connectivity between the amygdala and mPFC

Given that *OXTR*m was positively associated with structural connectivity between the amygdala and the mPFC and negatively associated with functional connectivity, we tested whether there was an inverse association between structural and functional connectivity and if *OXTR*m mediated this association. As hypothesized within the top-down regulatory framework, the number of diffusion tractography streamlines was negatively associated with amygdala–mPFC functional connectivity while controlling for age, sex, parent education, and brain volume (*B* = −0.072, *P* < 0.05, *η*_*p*_^*2*^ = 0.07). Using a bias-corrected bootstrapping procedure with 5000 samples [50], we observed a significant indirect effect of *OXTR*m mediating the association between structural and functional connectivity (*B* = −0.043, *P* = 0.01).

### Amygdala–mPFC functional connectivity and *OXTR* methylation predict internalizing behaviors 2 years later

**Fig. 3:**
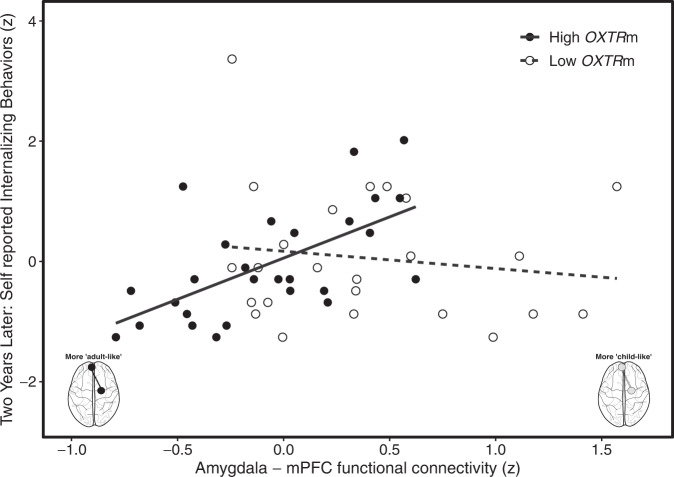
Amygdala–mPFC functional connectivity and *OXTR*m are predictive of children’s self-reported internalizing behaviors 2 years later. For visualization purposes, plotted is the raw data for the interaction of functional connectivity and a median split of *OXTR*m. The full Bayesian model utilized continuous variables. Individuals with higher *OXTR*m (black dashed line) are more sensitive to the association between amygdala–mPFC functional connectivity and internalizing behaviors compared to individuals with low methylation (solid line).

We fit the full model for self-reported internalizing behaviors with normalized predictors to estimate population effects for amygdala–mPFC functional connectivity, *OXTR*m, amygdala–mPFC streamlines, total brain volume, sex, age, parent education, as well as the interaction between amygdala–mPFC functional connectivity and *OXTR*m using a Bayesian general linear model (see “Materials and methods”). The central hypotheses of interest are the interaction term and the amygdala–mPFC streamlines. The other terms were included as covariate adjustments typically used in modeling internalizing behaviors. Using leave-one-out cross-validation [51], the results suggest that the interaction term and main effects between amygdala–mPFC functional connectivity and *OXTR*m improved model fit (see Table 1). The 95% credible interval for the interaction parameter did not overlap with 0 or negative values, which suggests that the effect is positive and is analogous to the notion of Frequentist significance (see Table 2). Individuals with higher *OXTR*m levels exhibited a positive relationship between amygdala–mPFC functional connectivity and internalizing behaviors. This relationship became more negative for individuals with lower *OXTR*m levels. See Fig. 3 for visualization of the modeled interaction and posterior density distribution. Note that the overall pattern of results is the same when the covariates are not included, as well as when using the Frequentist approach.

**Table 1:** Leave-one-out model comparison results suggest the full model, including amygdala–mPFC functional connectivity and *OXTR*m fits best.

Model	ELPD difference	ELPD SE	Bayesian *R*^*2*^	Lower 95% CI	Upper 95% CI
Full model	0.00	0.00	0.41	0.22	0.55
No functional connectivity or *OXTR*m	−3.28	4.84	0.10	0.02	0.24
No functional connectivity x *OXTR*m interaction	−4.89	4.57	0.14	0.03	0.30
No functional connectivity	−5.00	4.77	0.13	0.03	0.28
No *OXTR*m	−6.31	3.97	0.24	0.06	0.42

The expected log pointwise predictive density (ELPD) estimates the generalizability of the model for new data, and the difference is relative to the best model. Models are ranked from top (better) to bottom (worse) in terms of fit.

**Table 2:** Population parameter estimates and uncertainty for full model predicting self-reported internalizing behaviors at the 2-year follow-up.

Parameter	β coefficient	Lower 95% CI	Upper 95% CI
Fixed			
Intercept	8.14	6.82	9.47
Amygdala–mPFC functional connectivity	2.82	1.08	4.70
* OXTR*m	2.04	0.45	3.68
Amygdala–mPFC streamlines	1.15	−0.35	2.72
Total brain volume	−1.03	−2.85	0.77
Sex	−1.76	−3.50	−0.03
Age (months)	0.58	−0.73	1.88
Parent education	−0.11	−1.31	1.12
Amygdala–mPFC functional connectivity X *OXTR*m	2.10	0.92	3.32
Skew normal			
σ	3.96	3.13	5.08
α	1.26	−4.63	6.90

## Discussion

In this study, we identified a novel association between the endogenous oxytocin system and differential maturation of amygdala–mPFC connectivity that in turn is associated with children’s internalizing behaviors 2 years later. We found that children with higher levels of *OXTR*m had a more negative and adult-like amygdala–mPFC functional connectivity profile, and this functional connectivity profile was associated with better concurrent self-regulation skills. In addition, we find that *OXTR*m is positively associated with the number of streamlines between the amygdala and the mPFC, providing structural evidence to support the hypothesized top-down model of emotion regulation, specifically that the mPFC downregulates amygdala reactivity in mature connectivity networks [18]. Lastly, we find that children with higher levels of *OXTR*m and more negative, adult-like amygdala–mPFC functional connectivity report fewer internalizing behaviors 2 years later. These results provide compelling evidence to suggest that epigenetic modification within *OXTR* may be a biological marker of differential maturation of amygdala–mPFC functional connectivity and socio-emotional development.

Previous research has demonstrated a cross-sectional developmental shift in amygdala–mPFC functional connectivity that is in part influenced by early-life stress, leading to accelerated development of this functional circuit [14, 18, 28, 52]. We hypothesized that these changes are associated with epigenetic regulation of the endogenous oxytocin system. Specifically, *OXTRm* is known to be dynamic during development [26, 30] and influenced by the amount of maternal engagement in early life [30]. While we did not measure maternal engagement in this study, using this framework, we propose a model in which higher levels of *OXTRm* are associated with *less* maternal engagement and *more* precocious maturation of the amygdala–mPFC circuit. This proposed model is congruent with the stress acceleration hypothesis in that premature development is an adaptive mechanism that may benefit children and manifest in better emotion regulation functioning in a stressful environment [17]. Future longitudinal studies should specifically test this proposed model in and include a measure of the child’s social environment and longitudinal changes in amygdala–mPFC connectivity.

Consistent with our proposed model, we show that higher levels of *OXTR*m were associated with more mature patterns of amygdala–mPFC functional connectivity in children, irrespective of chronological age. These findings complement and extend previous research describing early maturation of this circuit in previously institutionalized youth [18], and indicate that natural variation in *OXTR*m may serve as a readout for the social environment. In addition, more negative, adult-like functional connectivity between the amygdala and mPFC was associated with greater self-control in social situations, demonstrating a behavioral benefit of early neural maturation. Specifically, amygdala–frontal circuitry is hypothesized to be prematurely activated in harsh environments to support affective regulation, while epigenetic regulation of *OXTR* is hypothesized to blunt the salience of stressful social environments [53]. It is plausible that both neural circuits and *OXTR*m are influenced by a stressful environment, and may influence each other bidirectionally to promote adaptive functioning.

In addition to the functional changes in the amygdala–mPFC connectivity, we also provide novel evidence for structural differences in this circuitry. Specifically, we find that higher *OXTRm* is associated with greater structural connectivity between the right amygdala and left mPFC. Previous research has shown white matter integrity, measured by fractional anisotropy, increases with age in the uncinate fasciculus, a primary structural connection between the amygdala and prefrontal cortex [54]. Moreover, increased white matter integrity in the uncinate fasciculus is associated with a decrease in amygdala reactivity to emotional faces, suggesting more efficient mPFC regulation of the amygdala [54]. Furthermore, structural connectivity strength between the striatum and the prefrontal cortex increases with age and is negatively associated with both functional connectivity between these regions during a reward task and with impatient behavior [55]. The positive association we find between *OXTR*m and streamlines between the amygdala and mPFC provides structural evidence to support the role of the endogenous oxytocin system in the hypothesized top-down model of emotion regulation. Specifically, our results support the hypothesis that greater structural connectivity between mPFC and amygdala is associated with efficient mPFC downregulation of amygdala reactivity in mature connectivity networks [18], and this association between structural and functional connectivity is mediated by DNA methylation of *OXTR*.

This study aimed to identify neurobiological mechanisms that contribute to individual differences in psychopathology. While most prior work has focused on deficit models, attention should also be paid to the stress-adapted skills developed in harsh environments [56]. Accelerated maturation of this neural circuitry is usually adaptive at the time and can lead to a reduction in anxiety [18] and may facilitate resilience. We find that both amygdala–mPFC functional connectivity and *OXTR*m interact to significantly predict children’s internalizing behaviors, such that children with higher levels of *OXTR*m are more sensitive to the association between amygdala–mPFC functional connectivity and internalizing symptoms. Specifically, children with lower levels of *OXTR*m and a more positive, child-like functional connectivity profile reported more internalizing behaviors, while children with higher levels of *OXTR*m and a more negative, adult-like functional connectivity profile reported fewer internalizing behaviors. These findings suggest that *OXTR*m and early maturation of amygdala–mPFC circuitry are indeed adaptive and may buffer internalizing symptomology in early adolescence.

Evidence of accelerated maturation of amygdala–mPFC connectivity is largely supported by studies examining typically raised children compared to previously institutionalized children or children with a history of early-life stress. Here, we examined how natural variability within the environment and endogenous oxytocin system may impact the neural maturation of amygdala–mPFC connectivity in typically raised children. Our findings are consistent with previous literature suggesting that even without extreme circumstances of adversity, variation in normative social environments is still relevant for neurobiological and behavioral outcomes. However, it is important to consider that the current study was restricted to self-identified Caucasian Americans due to the fact that methylation levels have been shown to differ as a function of ethnic groups [57]. In addition, while parent education varied from high school diploma to professional degrees in our sample, the distribution skewed left, suggesting that the children in our sample mostly all come from highly educated households. Future studies should explore the associations between *OXTR*m and neural maturation in more diverse ethnic and socio-economic populations.

An additional important point in this longitudinal study to consider is the onset of the COVID-19 pandemic. Since all neuroimaging and saliva samples were collected prior to the onset of the COVID-19 pandemic and all follow-up data collection on internalizing symptoms 2 years later was collected during the pandemic, this study was uniquely poised to examine the association of neurobiological indexed early maturation on psychopathology during a global pandemic. While it is important to note that the effects of the COVID-19 pandemic are vast and varied for individuals [58], given the small sample size retained at the second timepoint, it would be difficult to control for all possible variability in the children’s environment as a result of the pandemic. However, despite specific and individual differences in environmental changes brought on by the pandemic, all children experienced some form of disruption to their routine social environment that required adaptation.

While previous work has associated changes in *OXTR*m with the social environment in human infants [30] and animal models [26], it is unknown if *OXTR*m is dynamic throughout childhood. Our approach is limited in that we cannot address the bidirectional influences of the social environment and neural development on the epigenetic regulation of *OXTR* or vice versa [59]. This study lacked assessment of childhood environment or caregiver relationships at the initial timepoint of data collection. Future studies should include direct assessment of child-caregiver relationships to provide more insight into how parental care may influence the overall neurobiological model of children’s development of emotion regulation and psychopathology.

Taken together, our findings provide the first evidence for the role of *OXTR*m in differential amygdala–mPFC circuitry maturation both functionally and structurally. The findings support our hypothesis that higher *OXTR*m in children is associated with a more adult-like neural profile, which in turn is associated with better socio-emotional behaviors. While we are limited in the causal inferences that can be drawn from the current study, previous research suggests that the social environment, primarily parental care, influences the methylation state of *OXTR*. Synthesizing the current findings suggests that *OXTR*m may be a mechanistic marker of differential neural maturation typically associated with variation in parental care. Lastly, *OXTR*m moderates the association between amygdala–mPFC functional connectivity and internalizing symptoms during a stressful life event, suggesting that epigenetic regulation of *OXTR* may serve as an adaptive mechanism in calibrating to one’s social environment.

## Supplementary information

Supplemental Material

Figure S2
