# Supplementary material for: An epigenetic mechanism for differential maturation of amygdala–prefrontal connectivity in childhood socio-emotional development
Source: Transl Psychiatry. 2023 Mar 13;13:91. doi: 10.1038/s41398-023-02380-y (PMC10009823; doi:10.1038/s41398-023-02380-y)
Supplement: Supplementary file 1 — Supplemental Material [file 41398_2023_2380_MOESM1_ESM.docx]

**Supplementary Material**

MATERIALS AND METHODS

**Epigeneotyping**

Two hundred nanograms of DNA were subjected to bisulfite treatment (Kit MECOV50, Invitrogen, Carlsbad, CA), which converts non-methylated cytosines to uracil and leaves methylated cytosines unmodified for downstream sequencing. A 116-base pair region of *OXTR* containing CpG site -934 was amplified via polymerase chain reaction (PCR) using 20 nanograms of bisulfite-converted DNA as a template, 0.2 µM primers TSL101F (5′-TTGAGTTTTGGATTTAGATAATTAAGGATT-3′) TSL101R (5′-biotin-AATAAAATACCTCCCACTCCTTATTCCTAA-3′ ) and using Pyromark PCR kits (Qiagen, Hilden, Germany). Samples were amplified in triplicate with the following cycling conditions: [Step 1: (95°C/15 min)/1 cycle, Step 2: (94°C/30 s, 56°C/30 s, 72°C/30 s)/50 cycles, Step 3: (72°C/10 min)/1 cycle, Step 4: 4°C hold]. Each PCR plate contained methylation standards (0% and 100% methylated), a positive control, and negative controls from bisulfite conversion and PCR. PCR amplification of a 116 bp product was confirmed by gel electrophoresis. DNA methylation for each sample was quantified by pyrosequencing (PyroMark Q24, Qiagen) using primer TSL101S (5′-AGAAGTTATTTTATAATTTTT-3′) and Pyromark Gold Q24 Reagents (Qiagen, Hilden, Germany). DNA collection for three samples was insufficient to obtain methylation levels. Interquartile outlier detection was used to identify three samples with high replicate variability. These samples were rerun to estimate deviant values and replicates that caused deviation for these samples were removed. Average mean deviation within replicates was ±1.90%. Reported epigenotypes are an average of the three replicates.

**Imaging Parameters**

T1-weighted high-resolution structural images were acquired using Siemens’ magnetization-prepared rapid-acquired gradient echo (MPRAGE) pulse sequence with the following specifications: echo time (TE) = 2.98ms; repetition time (TR) = 2300ms; flip angle (FA) = 9°; image matrix = 240 mm × 256 mm; slice thickness = 1 mm; 208 slices. Whole-brain functional images were acquired using the same parameters as the Adolescent Brain Cognitive Development protocol^61^. The specifications for the T2* weighted echo planar imaging (EPI) sequence sensitive to BOLD contrast were the following: TE = 30 ms; TR = 800 ms; FA = 52°; image matrix = 90 mm x 90 mm; slice thickness = 2.4 mm; slice gap = 2.4 mm; 552 slices. Diffusion-weighted images were acquired with an isotropic voxel size of 1.7×1.7×1.7mm^3^, TE=70ms and TR=2900ms; using a multi-shell protocol, 10 b=0 images and 64 gradient directions were collected at both b=1500s/mm^2^ and b=3000s/mm^2^.

**fMRI Preprocessing**

All data were preprocessed using the Configurable Pipeline for the Analysis of Connectomes (C-PAC version 1.6.0, [http://fcp-indi.github.io](http://fcp-indi.github.io/)). C-PAC is an open-source, configurable pipeline for the automated preprocessing and analysis of fMRI data^62^. C-PAC is a software implemented in Python that integrates tools from AFNI^63^, FSL^64^, and ANTS^65^ with custom tools, using the Nipype^66^ pipelining library, to achieve high-throughput processing on high performance computing systems.

Skull-stripped images were resampled to RPI orientation and then a non-linear transform between images and a 2mm MNI brain-only template (FSL^64^) was calculated using ANTs^65^. The skull-stripped images were additionally segmented into WM, GM, and CSF using FSLs FAST tool^67^. A WM mask was calculated by applying a 0.95 threshold to the resulting WM probability map and multiplying the result by a WM prior map (avg152T1_white_bin.nii.gz - distributed with FSL) that was transformed into individual space using the inverse of the linear transforms previously calculated during the ANTs procedure. A CSF mask was calculated by applying a 0.95 threshold to the resulting CSF probability map and multiplying the result by a ventricle map derived from the Harvard-Oxford atlas distributed with FSL^68^. The thresholds were chosen, and the priors were used to avoid overlap with grey matter.

Functional preprocessing began with resampling the data to RPI orientation, and slice timing correction. Next, motion correction was performed using a two-stage approach in which the images were first coregistered to the mean fMRI and then a new mean was calculated and used as the target for a second coregistration (AFNI 3dvolreg^69^). Nuisance variable regression was performed on motion corrected data using a 2nd order polynomial, band pass filtering for resting state between 0.01 Hz and 0.1 Hz, a 24-regressor model of motion^70^, nuisance signals obtained from white matter (CompCor^71^) and mean CSF signal, and mean global signal. WM and CSF signals were extracted using the previously described masks after transforming the fMRI data to match them in 2mm space using the inverse of the linear fMRI-sMRI transform. Nuisance variable regression residuals were written into MNI space at 2.4mm resolution and subsequently smoothed using a 6mm FWHM kernel. Time courses from the region of interest, right amygdala, were extracted for each subject and analyzed with dual regression.

The fMRI data reported were normalized to the standard MNI adult template but the same preprocessing pipeline was carried out using the 4.5 – 7.5 year old template from the MRI Study of Normal Brain Development (NIHPD)^72^ and the right amygdala seed from the Haskins Pediatric Atlas^73^ as a sensitivity check. This template was chosen as the younger age range is more likely to differ from adult brains. There were no appreciable differences in the main findings utilizing the pediatric template and atlas.

**Diffusion Analysis**

Diffusion images were preprocessed to correct for standard artifacts which included removal of thermal noise^74^, correction for Gibbs ringing^75^, susceptibility distortions^76^, subject motion^77^, and eddy currents^78^.

For a full description of preprocessing steps as well as reliability of metrics derived from this diffusion processing pipeline see Newman et., al 2020^39^. The white matter fiber orientation distribution (FOD) was then resolved at the voxel-wise level by processing the outermost b-value shell (b=3000s/mm^2^) using single-shell constrained spherical deconvolution, a technique to separate directional axonal signal from intracellular and extracellular isotropic diffusion^40^. Probabilistic tractography was performed by applying the iFOD2 algorithm which propagates streamlines between voxels based on the direction and amplitude of the underlying FOD^79^. Seeding of streamlines was performed by randomly selecting voxels within a whole brain mask created by co-registering using ANTs each subject’s skull-stripped T1 output from Freesurfer^65,80^. Streamlines were seeded and generated until 10,000,000 tracts were created that were each longer than 2.6mm without terminating. These streamlines were then pruned to 2,000,000 total tracts using spherical-deconvolution informed filtering of tractograms (SIFT), which ties the number of streamlines in each voxel to the magnitude of the underlying FOD^41^. This process matches the randomly generated streamlines to the underlying anatomically derived signal and prevents a biologically implausible number of tracts from traversing the same voxel. A whole brain connectome was then created using the AAL atlas^42^ by assigning each terminal end of each tract to a corresponding nearest atlas ROI. The output was a matrix where each value corresponded to the number of tracts connecting each region from the atlas. As all subjects provide the same number of total streamlines the number of tracts connecting any two regions can be inferred to be a relatively stronger/weaker axonal connection between those regions compared to other subjects. ROIs were selected and pooled if the ROI overlapped the search region defined in the fMRI analysis giving a final ROI in the left frontal cortex composed of AAL regions Superior Frontal cortex, Superior Orbital Frontal cortex, Middle Frontal cortex, Superior Medial Frontal cortex, and Orbital Medial Frontal cortex. The total number of streamlines with one end assigned to either the right or left amygdala and the other end assigned to one of the frontal ROIs were taken from the whole brain connectomes for each participant for use in analysis.

RESULTS

*OXTR* DNA methylation values were normally distributed and a Shapiro-Wilk test showed no significant departure from normality (*W* = 0.98, *p* = 0.313). There were no significant associations of *OXTR*m with demographic control variables including child age (*r*(57)=0.11, *p* = 0.408), sex (*t*(57) = -0.78, *p* = 0.441), or parent education (*r*(57)=0.07, *p* = 0.594).

Self-control scores ranged from 3-20 (*M* = 11.96, *SD* = 3.80) and there was no significant departure from normality (*W* = 0.97, *p* = 0.247). There was no significant association between *OXTR*m and self-control ((*r*(50)=0.08, *p* = 0.571) or with any of the demographic control variables of age, sex, or parent education (all *p* > 0.05). At the two-year follow-up visit, child reported internalizing behavior scores ranged from 1–18 with a positive skew (*W* = 0.93, *p* = 0.011). Externalizing behavior scores were similar and ranged from 0-19 with a positive skew (*W* = 0.95, *p* = 0.047).

**Table S1.** Descriptive statistics of demographic and scale variables

| **Variable** | **N** | **Mean** | **SD** | **Range** |
| --- | --- | --- | --- | --- |
| Age (years) | 57 | 8.17 | 1.61 | 5.87 – 11.82 |
| Sex | 57 | 32 F |  |  |
| Parent Education | 57 | 6.67 | 2.03 | 2 - 10 |
| *OXTR*m | 57 | 45.06 | 4.59 | 36.14 – 54.07 |
| SSIS Parent Reported Self Control | 50 | 11.96 | 3.8 | 3 - 20 |
| SSIS Child Reported Internalizing Behaviors (2-year follow-up) | 45 | 6.84 | 4.22 | 1 - 18 |

**Figure S1.**
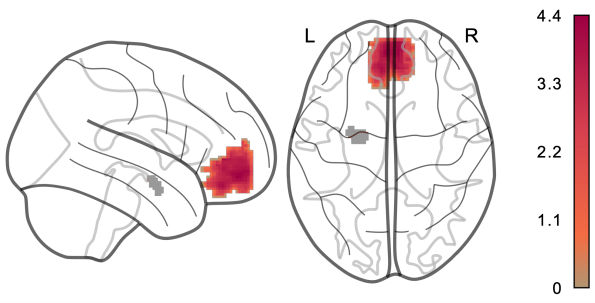


**Figure S1. Adults show reduced left amygdala-mPFC functional connectivity compared to children.** A) Z statistic map of voxels shows the significant main effect of age group (Children > Adults) in MNI space. The left amygdala seed region is depicted in gray.

**Figure S2.**

**Figure S2**. **Adults show higher *OXTR*m levels compared to children.** Methylation values are plotted for each individual in the full child cohort (*n* = 66, M = 44.36%) and the adult cohort (*n* = 31, M = 47.58%). *p<0.05.

61 Casey BJ, Cannonier T, Conley MI, Cohen AO, Barch DM, Heitzeg MM *et al.* The Adolescent Brain Cognitive Development (ABCD) study: Imaging acquisition across 21 sites. Dev. Cogn. Neurosci. 2018. doi:10.1016/j.dcn.2018.03.001.

62 Cameron C, Sharad S, Brian C, Ranjeet K, Satrajit G, Chaogan Y *et al.* Towards Automated Analysis of Connectomes: The Configurable Pipeline for the Analysis of Connectomes (C-PAC). *Front Neuroinform* 2013; **7**. doi:10.3389/CONF.FNINF.2013.09.00042/EVENT_ABSTRACT.

63 Cox RW. AFNI: Software for analysis and visualization of functional magnetic resonance neuroimages. *Comput Biomed Res* 1996. doi:10.1006/cbmr.1996.0014.

64 Smith SM, Jenkinson M, Woolrich MW, Beckmann CF, Behrens TEJ, Johansen-Berg H *et al.* Advances in functional and structural MR image analysis and implementation as FSL. In: *NeuroImage*. 2004 doi:10.1016/j.neuroimage.2004.07.051.

65 Avants B, Epstein C, Grossman M, Gee J. Symmetric diffeomorphic image registration with cross-correlation: evaluating automated labeling of elderly and neurodegenerative brain. *Med Image Anal* 2008; **12**: 26–41.

66 Gorgolewski K, Burns C, Madison C, Clark D, Halchenko Y, ML, Waskom M *et al.* Nipype: a flexible, lightweight and extensible neuroimaging data processing framework in python. *Front Neuroinform* 2011; **5**. doi:10.3389/FNINF.2011.00013.

67 Zhang Y, Brady M, Smith S. Segmentation of brain MR images through a hidden Markov random field model and the expectation-maximization algorithm. *IEEE Trans Med Imaging* 2001; **20**: 45–57.

68 Makris N, Goldstein J, Kennedy D, Hodge S, Caviness V, Faraone S *et al.* Decreased volume of left and total anterior insular lobule in schizophrenia. *Schizophr Res* 2006; **83**: 155–171.

69 Cox RW, Jesmanowicz A. Real-Time 3D Image Registration for Functional MRI. *Magn Reson Med* 1999; **42**: 1014–1018.

70 Friston K, Williams S, Howard R, Frackowiak R, Turner R. Movement-related effects in fMRI time-series. *Magn Reson Med* 1996; **35**: 346–355.

71 Behzadi Y, Restom K, Liau J, Liu T. A component based noise correction method (CompCor) for BOLD and perfusion based fMRI. *Neuroimage* 2007; **37**: 90–101.

72 Sanchez CE, Richards JE, Almli CR. Age-specific MRI templates for pediatric neuroimaging. *Dev Neuropsychol* 2012; **37**: 379–399.

73 Molfese PJ, Glen D, Mesite L, Cox RW, Hoeft F, Frost SJ *et al.* The Haskins pediatric atlas: a magnetic-resonance-imaging-based pediatric template and atlas. 2022; **51**: 628–639.

74 Veraart J, Novikov DS, Christiaens D, Ades-aron B, Sijbers J, Fieremans E. Denoising of diffusion MRI using random matrix theory. *Neuroimage* 2016; **142**: 394.

75 Kellner E, Dhital B, Kiselev VG, Reisert M. Gibbs-ringing artifact removal based on local subvoxel-shifts. *Magn Reson Med* 2016; **76**: 1574–1581.

76 Smith SM, Jenkinson M, Woolrich MW, Beckmann CF, Behrens TEJ, Johansen-Berg H *et al.* Advances in functional and structural MR image analysis and implementation as FSL. *Neuroimage* 2004; **23 Suppl 1**. doi:10.1016/J.NEUROIMAGE.2004.07.051.

77 Andersson JLR, Graham MS, Zsoldos E, Sotiropoulos SN. Incorporating outlier detection and replacement into a non-parametric framework for movement and distortion correction of diffusion MR images. *Neuroimage* 2016; **141**: 556–572.

78 Andersson JLR, Sotiropoulos SN. An integrated approach to correction for off-resonance effects and subject movement in diffusion MR imaging. *Neuroimage* 2016; **125**: 1063–1078.

79 Tournier J, Calamante F, Connelly A. Improved probabilistic streamlines tractography by 2 nd order integration over fibre orientation distributions. 2009.

80 Fischl B. FreeSurfer. *Neuroimage* 2012; **62**: 774–781.
