# Supplementary figures and images for: An epigenetic mechanism for differential maturation of amygdala–prefrontal connectivity in childhood socio-emotional development
Source: Transl Psychiatry. 2023 Mar 13;13:91. doi: 10.1038/s41398-023-02380-y (PMC10009823; doi:10.1038/s41398-023-02380-y)

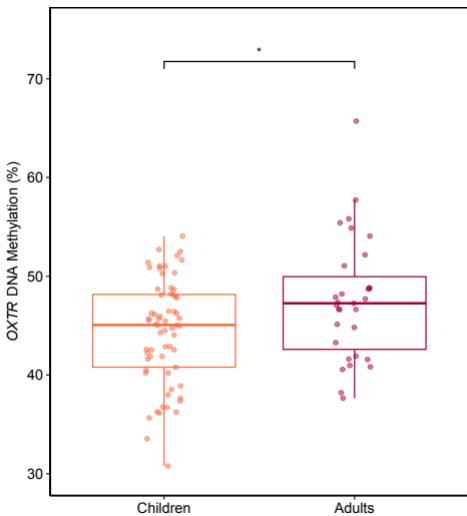

Supplement: Supplementary file 2 — Figure S2 [file 41398_2023_2380_MOESM2_ESM.pdf]
